# On the relationship between the specific heat enhancement of salt-based nanofluids and the ionic exchange capacity of nanoparticles

**DOI:** 10.1038/s41598-018-25945-0

**Published:** 2018-05-14

**Authors:** Rosa Mondragón, J. Enrique Juliá, Luis Cabedo, Nuria Navarrete

**Affiliations:** 1 Departamento de Ingeniería Mecánica y Construcción, Universitat Jaume I, 12071 Castellon de la Plana, Spain; 2 Polymers and Advanced Materials Group, Universitat Jaume I, 12071 Castellon de la Plana, Spain

## Abstract

Nanoparticles have been used in thermal applications to increase the specific heat of the molten salts used in Concentrated Solar Power plants for thermal energy storage. Although several mechanisms for abnormal enhancement have been proposed, they are still being investigated and more research is necessary. However, this nanoparticle-salt interaction can also be found in chemical applications in which nanoparticles have proved suitable to be used as an adsorbent for nitrate removal given their high specific surface, reactivity and ionic exchange capacity. In this work, the ionic exchange capacity mechanism for the nanoparticles functionalization phenomenon was evaluated. The ionic exchange capacity of silica and alumina nanoparticles dispersed in lithium, sodium and potassium nitrates was measured. Fourier-transform infrared spectroscopy tests confirmed the adsorption of nitrate ions on the nanoparticle surface. A relationship between the ionic exchange capacity of nanoparticles and the specific heat enhancement of doped molten salts was proposed for the first time.

## Introduction

The development of nanotechnology in recent decades has allowed nanoparticles to be used in different applications, such as thermal, optical, water treatment, pharmacology, development of new materials, etc. to improve the properties and efficiency of the involved processes.

In thermal sciences the use of inorganic molten salts (high temperature ionic liquids) for heat transfer systems (HTS) and thermal energy storage (TES) has received special attention, mainly for their use in concentrated solar power (CSP) plants^1^. Molten salts enable the storage of solar energy by balancing peak demand and revenue in such a volatile market as electric power generation. An important factor to select TES and heat transfer fluids (HTF) is that they have suitable thermal properties. The key property of TES working fluids is specific heat capacity as it indicates the sensible heat storage capacity of fluid. However, one major drawback of molten salts is their low specific heat.

Interest in the effect of nanoparticles on the specific heat of HTF and TES fluids appeared given the benefits reported in HTF when nanoparticles are added to increase thermal conductivity and the heat transfer coefficient^2,3^. In this case, according to the mixture rule, the specific heat of the nanofluid should decrease if nanoparticles with a lower specific heat than the base fluid are added. This theory agrees with the results obtained for molecular liquids, such as water, ethylene glycol, alcohols and thermal oils^4^. However for ionic liquid-based nanofluids (low-temperature organic salts and high-temperature inorganic molten salts), different results from those predicted by the theory were obtained.

Since the first works reported by Shin and Banerjee in 2011^5,6^, in which abnormal specific heat enhancement was achieved when titania and silica nanoparticles were added to mixtures of carbonate and chloride salts, several authors have focused on the specific heat enhancement of molten salts by adding nanoparticles^7–20^. In these works, silica, alumina, titania, carbon nanotubes and metallic nanoparticles were added to nitrate, carbonate and chloride salts to study the influence of particle size and morphology, solid content and the production process on specific heat enhancement. Molecular Dynamic simulations were also performed only considering the Lennard-Jones and Buckingham potentials with Coulombic interactions. Nonetheless, the obtained results were contradictory; an increment or decrease in specific heat can be achieved depending on the experimental conditions. All authors agree that the enhancement depends on the available specific surface of nanoparticles and the nanoparticle-salt interaction that occurs during nanofluid production, which is a key stage. Nevertheless, the exact mechanisms and theories for the enhanced thermo-physical properties of doped molten salts are not yet completely understood and are still being investigated^21^.

Initially, Shin and Banerjee^5,6^ proposed three possible mechanisms: (1) higher specific heat capacity for nanoparticles due to small particle size; (2) interfacial thermal resistance between nanoparticles and the surrounding liquid molecules that acts as additional thermal storage; and (3) formation of a semi-solid layer on the surface of solid particles with higher thermal properties than bulk liquid. Although the third mechanism is the most widely accepted one, the nanolayer concept fails to predict the experimental findings^2^. A mesolayer concept supports the idea that the effect of nanoparticles on the base fluid should be a much more wider-ranging one than previously assumed. An interacting mesolayer model has also been proposed to explain the specific heat maximum observed in several experimental studies. Finally, in low-temperature organic salts (also known as Ionic Liquids, IL, or IoNanofluids), the presence of a nanostructure within the bulk liquid and the interphase has been observed^4^. The interfacial structure results from the discrete layering of ions observed at a solid surface, which have preferred orientations, electrostatic interactions, dispersion forces and solvophobicity. The nature of the solid surface influences the ordering and composition of the surface layer via its surface charge, generated by the dissociation of surface groups, surface dielectric properties, and hydrophobicity or hydrophilicity. This interfacial structure of ordered layers of ions is the result of the nanoparticle-salt interactions that leads to the functionalization of nanoparticles, and could play an important role in the specific heat enhancement of molten salts (high temperature ionic liquids).

Apart from thermal applications, nanoparticle-salt interactions have already been studied and functionalization mechanisms have been proposed to be used in water treatment. In this area, oxide micro- and nanoparticles, nanoscale zerovalent material, carbon or graphene particles have been used as adsorbents to remove the nitrates, phosphates and cations of hazardous metals from water^22–26^. Adsorption is a surface phenomenon in which the physical adhesion of adsorbate occurs via the ionic and covalent interactions between that adsorbate and the functional groups present on the adsorbent surface. The benefits of using nanomaterials derive from their large surface area for adsorption and enhanced reactivity, where nanoadsorption is more efficient.

Physico-chemical conditions also influence the removal efficiency. The effect of contact time, adsorbent dosage, presence of other interfering ions (sulphates, ammonium), initial ion concentration, temperature and sonication time has been analysed in the literature to obtain the optimal conditions for each system. The nanoparticulate adsorbent dosage is important and each nanoparticle has an optimum value. Any concentration above that optimum dose may lead to reduced exposure of the active sites per unit mass due to agglomeration. Below the optimum value, an increasing amount of the adsorbent dose leads to more unsaturated active sites showcasing a greater removal extent.

**Figure 1:**
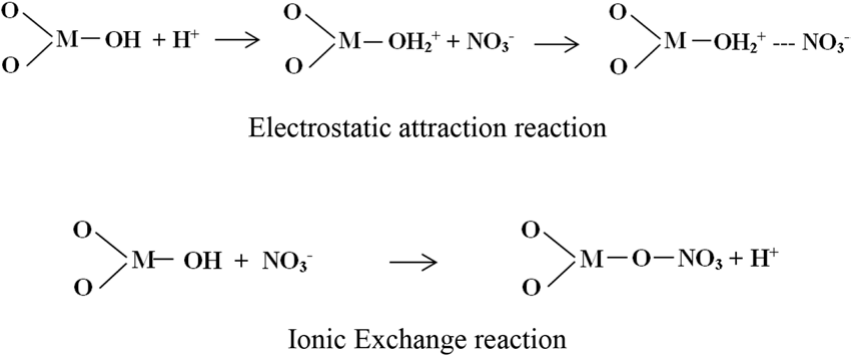
Functionalization mechanisms for oxide nanoparticles in nitrate salts.

Another important factor is the pH value. Changes in pH influence the adsorption mechanism^27–29^. Specific and/or non-specific adsorption is proposed to remove anionic contaminants. Non-specific adsorption involves coulombic forces, while specific adsorption involves ligand exchange reactions. Accordingly, two mechanisms were proposed (see Fig. 1). In the first one, metal oxides were activated with an acidic solution, and the available surface hydroxyl groups were protonated to form positive groups in order to provide a strong electrostatic interaction with anions. At low pH values,adsorption capacity was high. However as pH rises, the positive charge extent lowers to reach the zero value at the isoelectic point of the nanoparticle. In the second one, Fourier-transform infrared spectra (FTIR) after adsorption tests indicated new bonds that could form during the adsorption process due to anion exchange reactions.

The adsorption kinetics describes two phases^28,30^. The first one can be attributed to the most readily sorbing sites on the adsorbent surface. The second one can be attributed to very slow diffusion of the sorbate from the surface site to inner pores, and may be controlled by pore diffusion. The amount of sorbate adsorbed on the solid particle surface increases with time to reach a maximum value at the equilibrium time. The adsorption process is reversible and desorption can occur under alkali conditions. However, the saturated adsorbent cannot be completely regenerated, which indicates that physical forces may exist besides the electrostatic interaction during the adsorption course. The adsorption phenomenon has not been taken into account in the Molecular Dynamic simulations performed on salt-based nanofluids.

The ionic exchange capacity (IEC) of the nanoparticles used as the adsorbent has already been evaluated in ion-exchange membranes doped with oxide nanoparticles, used also in water treatment^31–34^. The influence of both the pH value and nanoparticle concentration on exchange efficiency was studied.

As the ionic exchange capacity of nanoadsorbents and the specific heat of salt-based nanofluids depend on both the available specific surface and nanoparticle-salt interaction, the relationship between both properties was analysed herein. The lithium, sodium and potassium nitrates doped with silica and alumina nanoparticles were tested. Fourier-transform infrared spectroscopy (FTIR) was used to confirm the adsorption of the nitrate ions on the nanoparticle surface and a possible mechanism that contributes specific heat enhancement was provided.

## Results

### Particle size distribution

**Figure 2:**
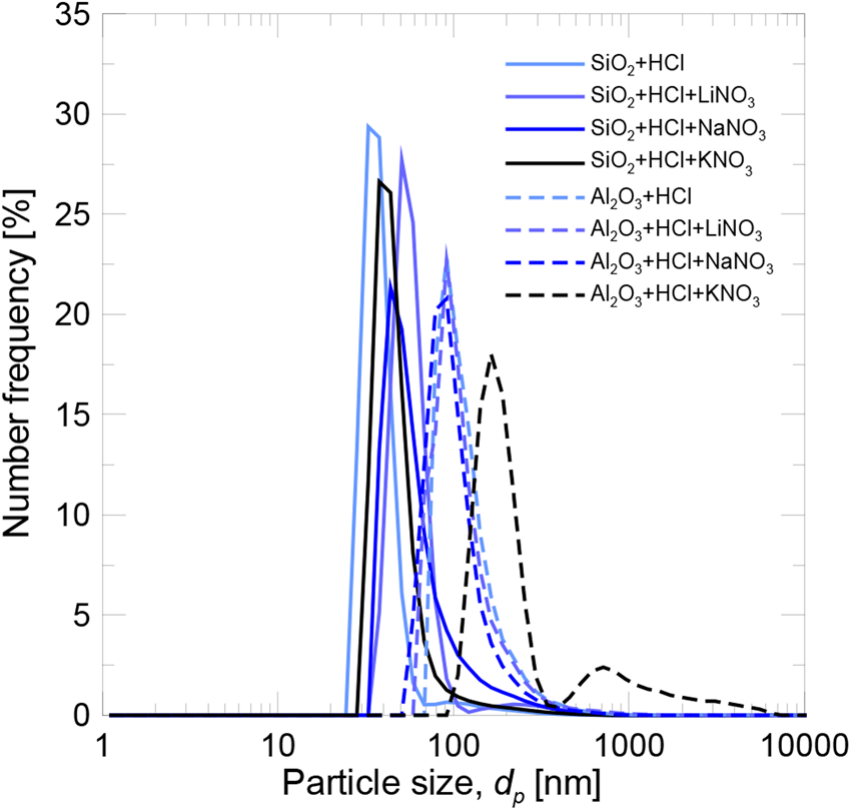
Particle size distribution in the aqueous solution.

The particle size distributions of the silica and alumina nanoparticles dispersed in aqueous solutions of HCl and nitrates are shown in Fig. 2. The diameter, below which 50% of particles were found, *d*_*p50*_, is shown in Table 1. Both nanoparticles agglomerate and bigger sized clusters than the primary particles are present. These clusters were those that interacted with the surrounding media and, hence, with the involved phenomena,and the final properties depended on their size.

**Table 1:** *pH* and particle size of aqueous solutions containing nanoparticles.

	*pH*	*d*_*p50*_ [nm]
SiO_2_+HCl	0.93	37.7
SiO_2_+HCl+LiNO_3_	1.06	56.7
SiO_2_+HCl+NaNO_3_	1.13	55.4
SiO_2_+HCl+KNO_3_	1.19	45.1
Al_2_O_3_+HCl	0.89	108
Al_2_O_3_+HCl+LiNO_3_	1.03	105
Al_2_O_3_+HCl+NaNO_3_	1.05	96.7
Al_2_O_3_+HCl+KNO_3_	1.13	188

For the silica nanoparticles, the achieved dispersion was similar in allsamples, the order of magnitude of the final size was the same (below 57 nm) and did not depend on the chemical composition of salt. Although the primary particle size for the alumina nanoparticleswas smaller, the final size of agglomerates was bigger (above 96 nm), and the available specific surface for the ionic exchange process was smaller. In this case, greater agglomeration was observed only for the KNO_3_ dissolution.

Table 1 shows the pH value of the samples immediately after sonication. Here the pH in them all was lower than that which corresponded to the isoelectric point.Therefore, the degree of dispersion achieved is the maximum possible and the samples are stable under these conditions.

### Ionic exchange capacity

**Figure 3:**
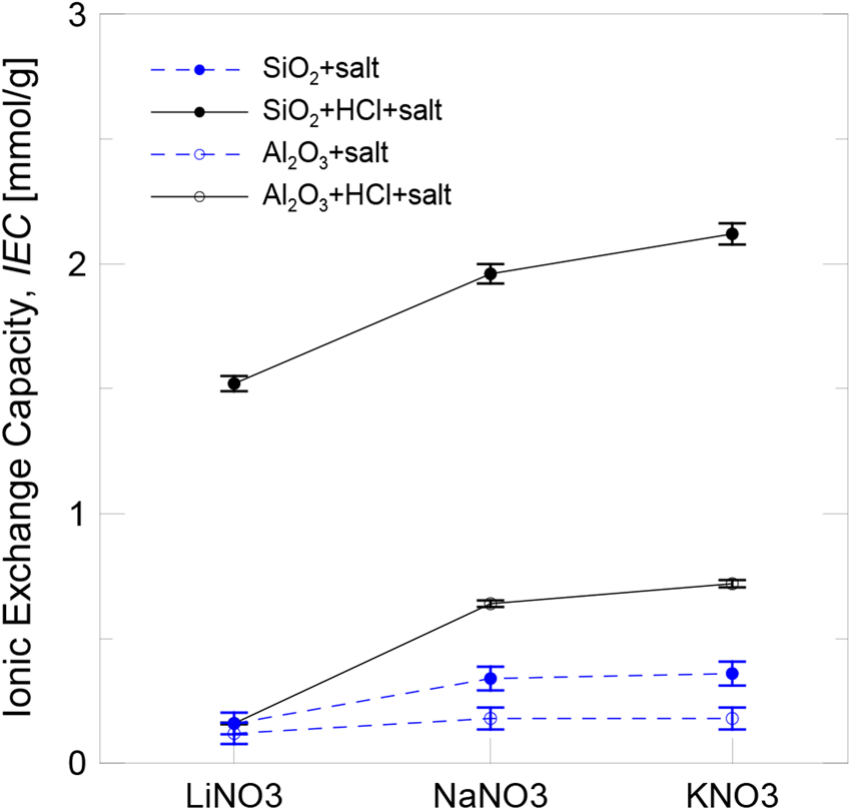
IEC of the nanoparticles in salt solution.

Figure 3 shows the results obtained for the IEC of the silica and alumina nanoparticles dispersed in salt solution following both the aforementioned methods: protonated by adding HCl and non-protonated. In all cases, the IEC of the silica nanoparticles was higher than that of the alumina nanoparticles. As the ionic exchange is a phenomenon that depends on nanoparticles surface, those with the lowest particle size and the largest available specific surface (silica) were the ones which provided a higher IEC. Despite nanoparticles not being previously protonated, they presented an IEC, althoughreduced. However,their IEC increased when they were protonated thanks to the addition of HCl. This means that acid activation provided a larger number of exchangeable H^+^on the nanoparticles surface and also contributed to the ionic exchange mechanism.

Regarding the effect of the chemical composition of the salt, it can be concluded that the IIEC increased with the size of the involved cation (Li^+^, Na^+^ and K^+^). According to the proposedionic exchange mechanism, H^+^ from the nanoparticle surface were displaced by nitrate functional groups, NO_3_^−^. This means that their affinity for nanoparticles was higher than for the cation when its size is increased.

### Chemical composition and functionalization of nanoparticles

The protonated silica and alumina nanoparticles dispersed in LiNO_3_, NaNO_3_ and KNO_3_ were used to prepare salt-based nanofluids to evaluate the change in chemical composition, as well as the relationship between IEC and the specific heat.

Changes in the chemical composition of nanoparticles were observed in the FTIR spectra. It was noteworthythat the functionalization process was a reversible one, and thatthe desorption of the adsorbed functional groups occurred when nanoparticles were suspended in water. Accordinglyin the isolation stage of nanoparticles, in which they were centrifuged in the presence of water to remove the salt content, the degree of functionalization lowered. Therefore, the obtained spectra could be used to determine the presence of ionic groups in nanoparticles, but not to quantify the real degree of functionalization of the nanoparticles dispersed in salt, which was always higher than that observed after isolation.

**Figure 4:**
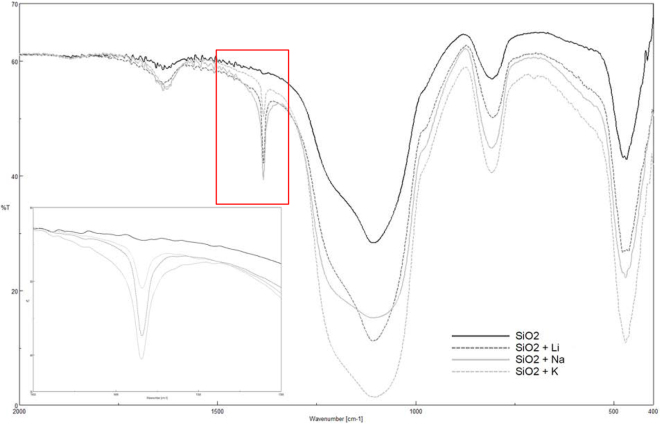
FTIR spectra for functionalized silica nanoparticles.

**Figure 5:**
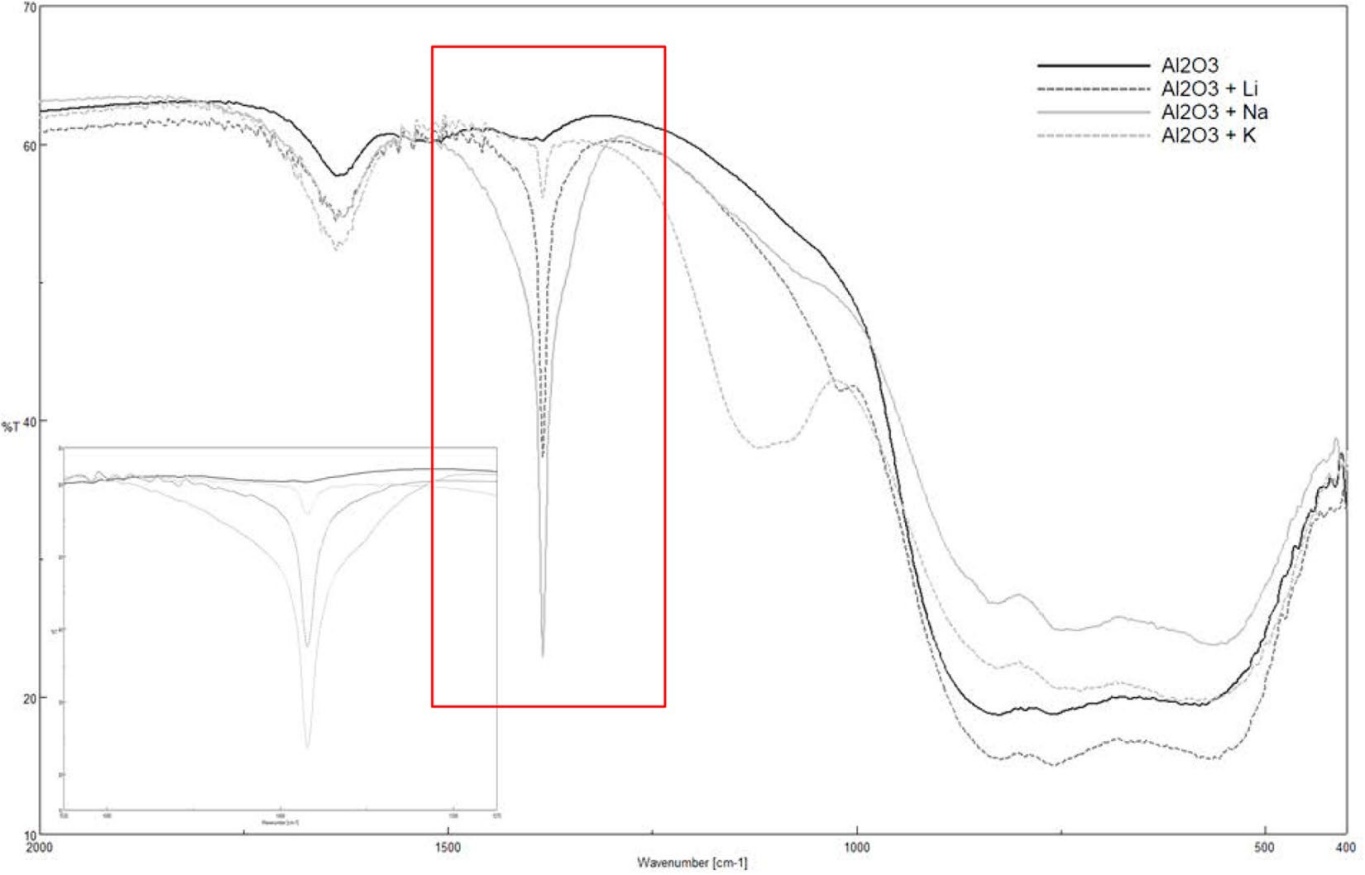
FTIR spectra for functionalized alumina nanoparticles.

**Figure 6:**
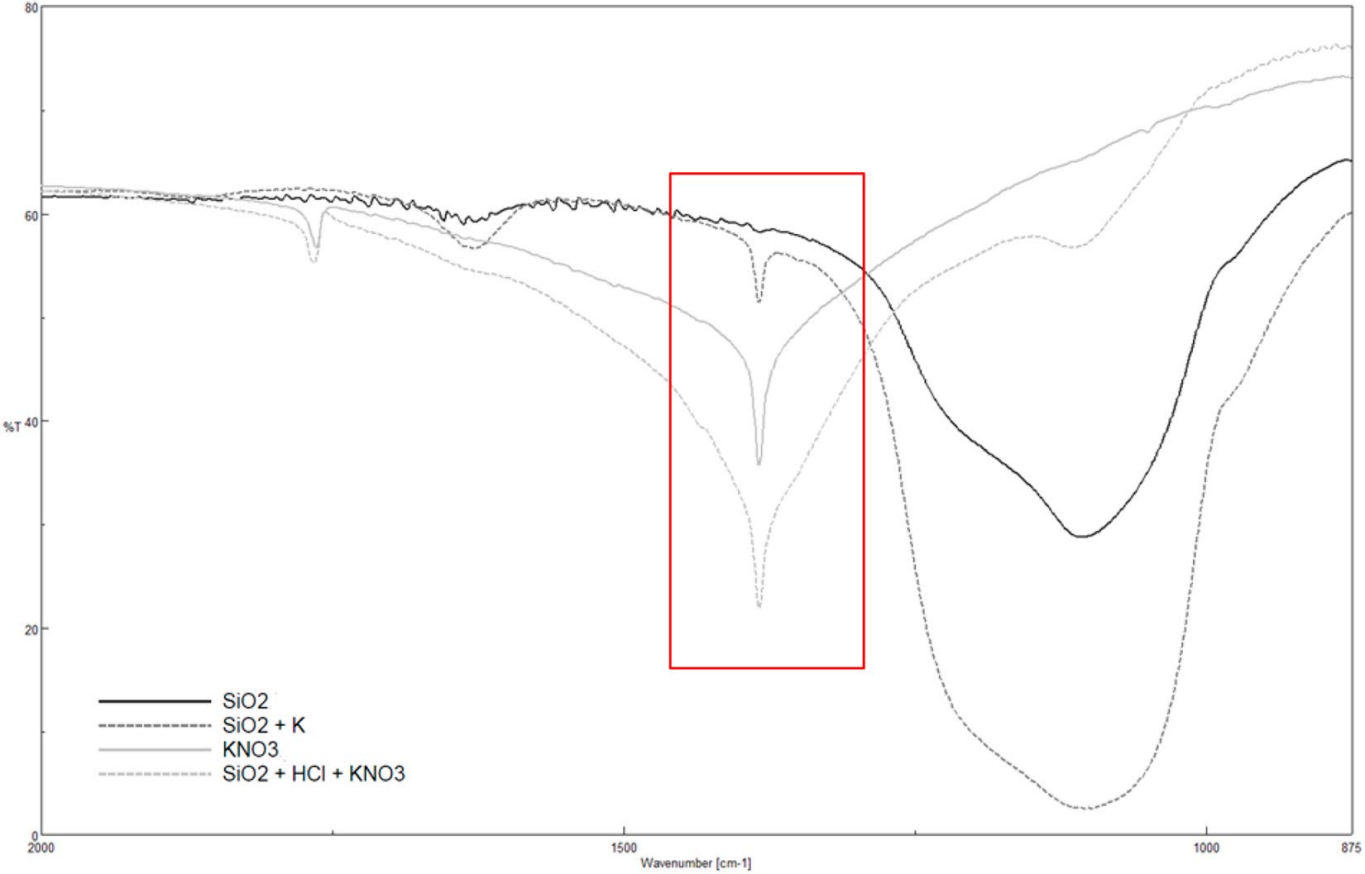
FTIR spectra for pure KNO_3_, KNO_3_ doped with silica and functionalized silica nanoparticles.

Figures 4 and 5 respectivelyshow the FTIR spectra for the silica and alumina nanoparticles functionalized with the lithium, sodium and potassium nitrates. For both nanoparticles, it can be observed that a new peak at 1.380 cm^−1^ appeared regardless of which nitrate was employed. This peak corresponded to the nitrate ion, NO_3_^−^, and it came from nitrate salt (see Fig. 6). According to the literature, the nitrate ion displays very strong absorption at 1.410–1.340 cm^−1^, which matches the results obtained in this work^35^.

These results, together with the ionic exchange capacity tests, confirmed that when oxide nanoparticles were surrounded by the nitrate functional groups from salt,these groups adsorbed onto the particle surface and H^+^were displaced via the mechanism proposed in Fig. 1.

### Specific heat capacity

**Figure 7:**
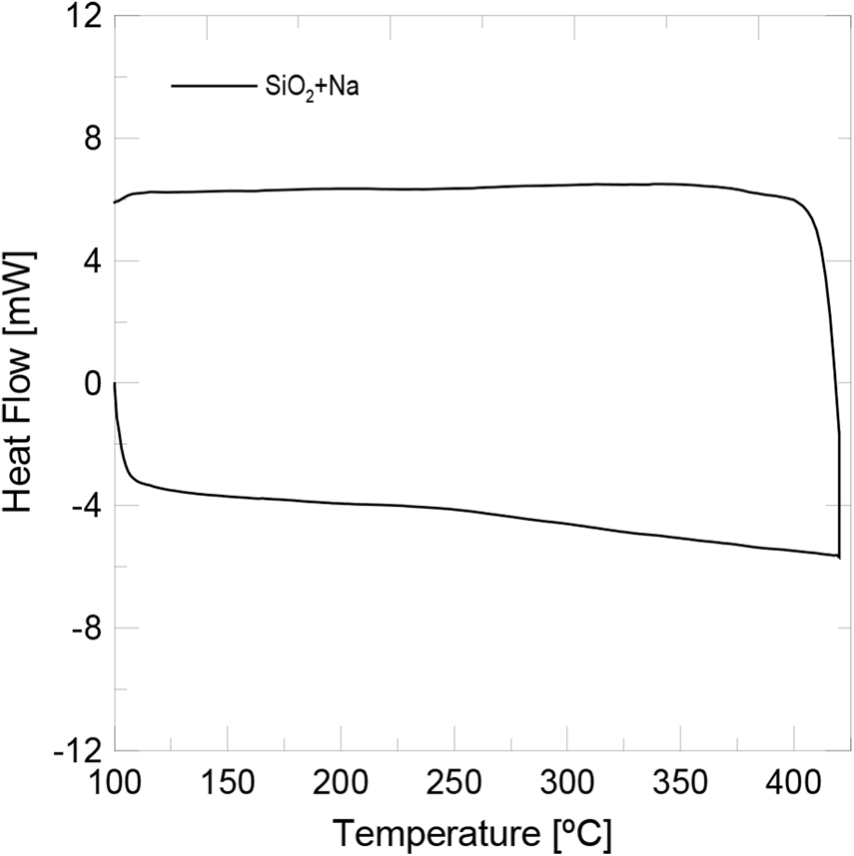
DSC curve for silica nanoparticles functionalized with NaNO_3_.

In order to check the absence of salt in the nanoparticles after being isolated and therefore the peak observed in FTIR spectrum is due to the functionalization, the silica nanoparticles functionalized with sodium nitrate were submitted to a DSC cycle, which was run from 100 °C to 400 °C at a heating rate of 20 °C/min. Figure 7 reveals that there were no peaks at the melting or crystallisation temperature of sodium nitrate (308 °C). Hence no salt was presentin nanoparticles.

**Figure 8:**
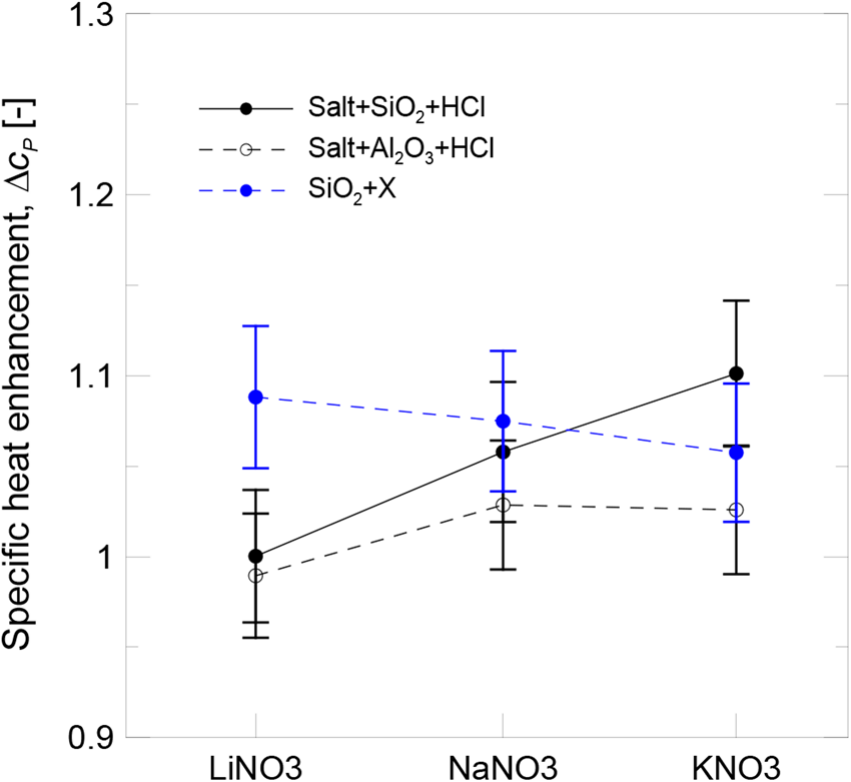
Specific heat enhancement.

The specific heat capacity of the silica nanoparticles functionalized with LiNO_3_, NaNO_3_ and KNO_3_ (XNO_3_) was measured at 400 °C and the results are shown in Fig. 8, which illustrates that specific heat increased in all the samples and that enhancement could be related to the intensity of the FTIR peak at 1.380 cm^−1^. As previously mentioned, since adsorption is a reversible process that depends on the solubility of the involvedions, the intensity of the peak and the degree of functionalization of the isolated nanoparticles after the dissolved salt was removed differed from the real ones in molten salt. Therefore,it was not possible to compare the evolution of the specific heat of the isolated nanoparticles with the results obtained for the salt-based nanofluids. Nevertheless,the sample with the smallest FTIR peak area also providedthe least specific heat enhancement. These results suggest that the adsorption of nitrate ions on the nanoparticle surface contributes to enhance the specific heat of the nanoparticles.

**Figure 9:**
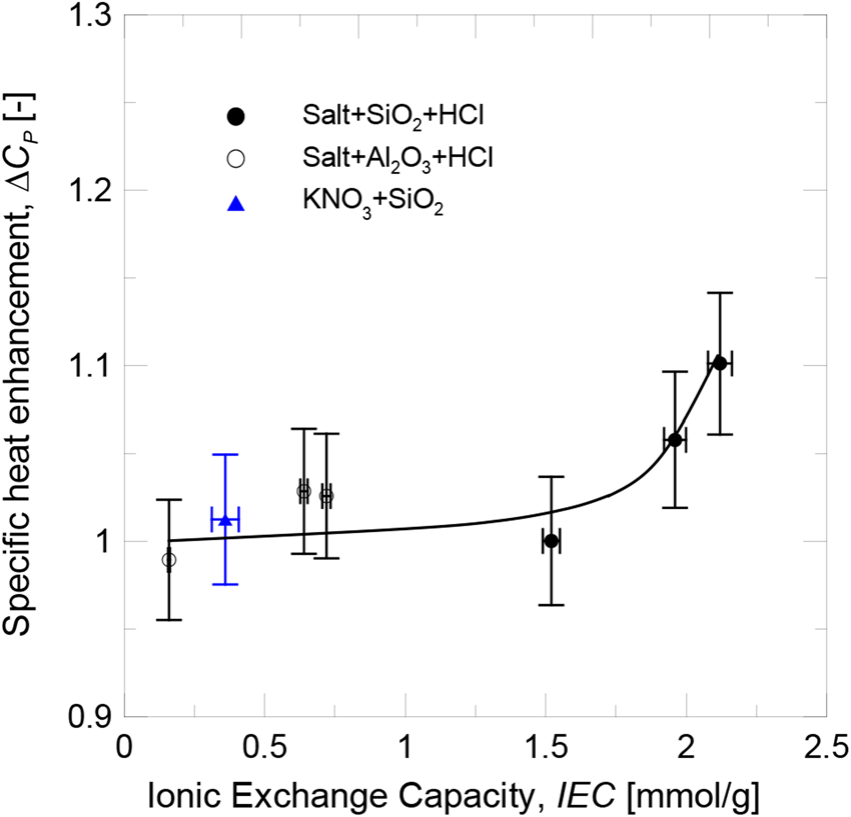
Evolution of specific heat enhancement with IEC.

The specific heat enhancement of the salt-based nanofluids at 400 °C, which contained the previously protonated silica and alumina nanoparticles, is also shown in Fig. 8. It can be observed the same trend obtained for IEC tests as shown in Fig. 3. The differences observed for the silica and alumina nanoparticles lay in the different particle size distribution and the specific surface available for the nanoparticle-salt interaction. The results agree with those found in the literature, and the silica nanoparticles provided greater specific heat enhancement than the alumina nanoparticles due to their smaller size and, thus,bigger specific surface. Regarding the influence of the chemical composition of the salt, the specific heat enhancement for both nanoparticles increased as the size of the involved cation (Li^+^, Na^+^ and K^+^) augmented. It can be deduced that the experimental conditions under which IEC became higher (small particle size and bigger cation size) were those that also presentedgreater specific heat enhancement. Therefore, the relationship between both variables was confirmed (see Fig. 9) and specific heat enhancement could be explained by taking into account the influence of the variables involved in the ionic exchange process.

It is noteworthy that all the samples were prepared with 1 M salt solutions with solid mass loads of nanoparticles which ranged from 1.9% to 2.8%, but the agglomerate size of each particle type remained almost constant. If the available specific surface was not affected, the influence of mass content could be negligible.

Besides, it has been reported in the literature^11,36^ that there exists a maximum specific heat enhancement for 1% of solid mass load approximately and then there is a decrease of the specific heat below the pure salt value for solid mass loads higher than 1.5% approximately. In this case, in spite of the high solid content tested a not expected specific heat enhancement was achieved for SiO_2_-KNO_3_ nanofluid (10.1%).

### Relationship between specific heat capacity and IEC

The evolution of the specific heat enhancement with the ionic exchange capacity of nanoparticles is shown in Fig. 9. The results for the salt-based nanofluids doped with the protonated silica and alumina nanoparticles are plotted together, and a relationship is seen between both variables, while two regions are distinguished. For low IEC values, the degree of functionalization of nanoparticles was not high enough to have a significant effect on the specific heat of nanofluids. However when the IEC increased beyond a threshold value,the specific heat enhancement increased due to the contribution of the highly functionalized nanoparticles.

These results confirm that an ionic exchange process occurred between the nanoparticle and the saltin the preparation stage in accordance with the proposedmechanism, which led to the formation of a new ordering and composition on the nanoparticle surface layer due to the adsorption of nitrate ions. The degree of functionalization of nanoparticles influenced the specific heat of the nanofluid and contributed to its enhancement.

In order to corroborate that the specific heat enhancement depended on the IEC of the nanoparticles, KNO_3_ doped with non-protonated silica was prepared. In this case, the IEC fell in the low values region (IEC = 0.36 mmol/g). As expected, the specific heat enhancement was also low (1.3%), unlike the value obtained for the protonated nanoparticles (10.1%, the maximum value achieved in this work). The results are also plotted in Fig. 9.

Finally, the effect of the nanoparticle concentration on the IEC and the specific heat enhancement was evaluated for 25 ml of KNO_3_ 1 M doped with 0.1 g of protonated silica (3.73 wt%). Results are shown in Table 2. It can be observed that the increase in the concentration lead to the agglomeration of the nanoparticles thus increasing the mean particle size and lowering the available specific surface. As a result the amount of H^+^ adsorbed during the protonation with HCl is also reduced and the final IEC and specific heat enhancement decrease with respect to the formulation at 1.92 wt%. When analyzing the influence of the solid content two opposite effect have to be taken into account. On the one hand, the increase in the solid content and the agglomeration of nanoparticles produce a reduction in their degree of functionalization. On the other hand, the amount of functionalized nanoparticles contributing to the specific heat is increased. Therefore, a commitment between IEC and mass content has to be established and more research on these effects is needed.

**Table 2:** Influence of solid content on IEC and specific heat enhancement for KNO_3_ doped with SiO_2_.

SiO_2_ mass load [%]	*pH*	*d*_*p50*_ [nm]	${{\boldsymbol{n}}}_{{{\boldsymbol{H}}}_{{\boldsymbol{acid}}}^{{\boldsymbol{+}}}}/{{\boldsymbol{m}}}_{{\boldsymbol{np}}}$[mmol/g]	*IEC* [mmol/g]	*Δc*_*P*_[−]
1.92	1.19	45.1	3.32	2.12	1.101
3.73	1.02	112.8	0.66	0.42	1.068

## Conclusions

This work found that if nanoparticles were dispersed in salt solutions or molten salts, they would interact with the nitrate ionspresent. The IEC tests showed the exchange of H^+^ by a functional group, while the FTIR tests confirmed the adsorption of nitrate groups on the nanoparticles surface. The ionic exchange mechanism was proposed and checked.

A qualitative relationship between the IEC of the nanoparticles and the specific heat enhancement of the molten salt-based nanofluids was established. Specific heat increased when IEC rose, mainly when a threshold value was achieved. Theconclusion drawn wasthat the presence of functionalized nanoparticles contributed to specific heat enhancement, and more research needs to be done to provide a more in-depth explanation of the specific heat enhancement mechanism.

As the IEC of the nanoparticles depends on the pH value, the dispersion degree and hence the available particle surface, the preparation stage is of great importance as was found in literature for specific heat studies. Also the chemical composition of the salt, the salt and nanoparticles concentrations, the nanoparticles size and shape and the temperature influence the IEC and can be optimized in order to get higher specific heat enhancements.

## Methods

### Sample preparation

Different nitrate salts were used to carry out the experiments: lithium nitrate (LiNO_3_purum ≥98%, Sigma-Aldrich, Inc.), sodium nitrate (NaNO_3_ Analytical grade ACS, Labkem) and potassium nitrate (KNO_3_ Analytical grade ACS, Labkem). Salts were doped with silica and alumina nanoparticles. Silica nanoparticles (99.8% purity) were purchased in powder form from Sigma-Aldrich. According to the manufacturer the average diameter of the primary particles is 12 nm. The isoelectric point for silica is found at pH = 2. Alumina nanoparticles (Gamma, 99.99% purity) were purchased from Nanostructured & Amorphous Materials, Inc. also in powder. According to the manufacturer the diameter of the primary particles is 10 nm. The isoelectric point for alumina is found at pH = 8.

Hydrochloric acid (HCl solution 1N, Scharlab, SL) and sodium hydroxide (NaOH granulated, synthesis grade >98%, Scharlab, SL) were used to prepare the dissolutions needed for the protonation and titration experiments. Phenolphthalein (solution 1% in ethanol, Scharlab, SL) was used as an indicator.

Salt-based nanofluids were prepared by means of the dissolution method. Firstly, 0.2 g of nanoparticles were dispersed in 100 ml of HCl_(aq)_ 0.1 M using an ultrasound probe (Sonopuls HD2200, Bandelin electronic GmbH & Co.) for 1 min. The sample was magnetically stirred (C-Mag HS7, IKA®-Werke GmbH & Co.) in order to be protonated. After 24 h, 100 ml of the corresponding salt solution 1 M were added and the sample was sonicated again for 1 min. The suspension was magnetically stirredfor 48 h. Finally, it was spread in Petri dishes to increase the available drying interphase and was dried in an oven at 100 °C for 3 h.

In order to isolate the nanoparticles after the nanofluid synthesis, part of the doped salt was redissolved in distilled water and centrifuged for 10 min at 4000 rpm in a centrifuge (model 2698, Nahita® - ICT, SL). The procedure was repeated 5 to 7 times to reduce the electrical conductivity of the water to values that came close to distilled water. This ensured full removal of salt content. Finally, the solid nanoparticles were dried in an oven at 100 °C.

Table 3 offers a description of all the samples, together with the corresponding ID used in this work to refer to them.

**Table 3:** Sample IDs and description.

Sample ID	Description
SiO_2_	Pure SiO_2_ nanoparticles (NPs)
SiO_2_+HCl	SiO_2_ NPs suspended in HCl solution
SiO_2_+HCl+LiNO_3_	SiO_2_ NPs suspended in mixture of HCl and LiNO_3_ solutions
SiO_2_+HCl+NaNO_3_	SiO_2_ NPs suspended in mixture of HCl and NaNO_3_ solutions
SiO_2_+HCl+KNO_3_	SiO_2_ NPs suspended in mixture of HCl and KNO_3_ solutions
SiO_2_+salt	SiO_2_ NPs suspended in a salt solution (salt: LiNO_3_/NaNO_3_/KNO_3_)
SiO_2_+HCl+salt	SiO_2_ NPs suspended in mixture of HCl and a salt solution (salt: LiNO_3_/NaNO_3_/KNO_3_)
SiO_2_+Li	SiO_2_ NPs after functionalization with LiNO_3_
SiO_2_+Na	SiO_2_ NPs after functionalization with NaNO_3_
SiO_2_+K	SiO_2_ NPs after functionalization with KNO_3_
SiO_2_+X	SiO_2_ NPs after functionalization with nitrate salt XNO_3_ (X: Li/Na/K)
Al_2_O_3_	Pure Al_2_O_3_nanoparticles (NPs)
Al_2_O_3_+HCl	SiO_2_ NPs suspended in HCl solution
Al_2_O_3_+HCl+LiNO_3_	Al_2_O_3_ NPs suspended in mixture of HCl and LiNO_3_ solutions
Al_2_O_3_+HCl+NaNO_3_	Al_2_O_3_ NPs suspended in mixture of HCl and NaNO_3_ solutions
Al_2_O_3_+HCl+KNO_3_	Al_2_O_3_ NPs suspended in mixture of HCl and KNO_3_ solutions
Al_2_O_3_+salt	Al_2_O_3_ NPs suspended in a salt solution (salt: LiNO_3_/NaNO_3_/KNO_3_)
Al_2_O_3_+HCl+salt	Al_2_O_3_ NPs suspended in mixture of HCl and a salt solution (salt: LiNO_3_/NaNO_3_/KNO_3_)
Al_2_O_3_+Li	Al_2_O_3_ NPs after functionalization with LiNO_3_
Al_2_O_3_+Na	Al_2_O_3_ NPs after functionalization with NaNO_3_
Al_2_O_3_+K	Al_2_O_3_ NPs after functionalization with KNO_3_
Salt+SiO_2_+HCl	Molten salt doped with SiO_2_ NPs previously protonated with HCl
Salt+Al_2_O_3_+HCl	Molten salt doped with Al_2_O_3_ NPs previously protonated with HCl
KNO_3_+SiO_2_	Molten KNO_3_ doped with SiO_2_ NPs

### Particle size distribution

The particle size provided by the manufacturer corresponds to the diameter of the primary nanoparticles. However, they form agglomerates of bigger size when they are suspended in the base fluid. The particle size distribution of the nanoparticles dispersed in the aqueous solutions was measured by means of Dynamic Light Scattering (DLS) using a ZetaSizer Nano ZS (Malvern Instruments, Ltd.). Tests were performed with a 173° scattering angle.

### Ionic exchange capacity

Ionic exchange capacity (IEC) is defined as the moles of ion exchanged group per gram of dry nanoparticle. In this work the IEC was measured by the titration method, by measuring the protons (H^+^) displaced by the ion exchanged groups of the salt solution. Two different methods based on those found in the literature for other materials were used to measure the IEC of the nanoparticles dispersed in a salt solution, both with and without previous protonation.

- In the first one, 0.05 g of nanoparticles were dispersed in 25 ml of HCl 0.1 M with an ultrasound probe for 1 min and the sample was magnetically stirred. After 24 h, it was titrated with NaOH 0.1 M. The moles of H^+^ adsorbed on the nanoparticle surface from the HCl solution (${{\rm{n}}}_{{{\rm{H}}}_{{\rm{acid}}}^{+}}$) were calculated by the following equation:
  1 $${n}_{{H}_{acid}^{+}}={V}_{HCl}\cdot {M}_{HCl}-{V}_{NaOH}\cdot {M}_{NaOH}$$
  where *V*_*HCl*_ and *M*_*HCl*_ are the volume and concentration of the initial HCl solution respectively, and *V*_*NaOH*_ and *M*_*NaOH*_ are the volume and concentration of the NaOH used in the titration respectively.
  In order to measure the IEC of the nanoparticles in the different salt solutions (LiNO_3_, NaNO_3_ and KNO_3_), the first step was repeated. After 24 h in HCl, 25 ml of the corresponding salt solution 1 M were added without removing the acid solution. The sample was sonicated again for 1 min and magnetically stirred for 48 h. Afterwards the nanofluid was titrated with NaOH 0.1 M. Finally, the IEC was calculated as follows:
  2 $${n}_{{H}_{salt}^{+}}={V}_{HCl}\cdot {M}_{HCl}-{V}_{NaOH}\cdot {M}_{NaOH}$$
  where ${n}_{{H}_{salt}^{+}}\,$are the moles of H^+^ that remained adsorbed on the nanoparticle surface after the salt interaction, *V*_*HCl*_ and *M*_*HCl*_ are the volume and concentration of the initial HCl solution respectively, and *V*_*NaOH*_ and *M*_*NaOH*_ are the volume and concentration of the NaOH used in the titration respectively.
  3 $$IEC=\frac{{n}_{{H}_{acid}^{+}}-{n}_{{H}_{salt}^{+}}}{{m}_{np}}$$
  where ${m}_{np}\,$is the mass of nanoparticles.
- In the second method, 0.05 g of nanoparticles were dispersed in 25 ml of the corresponding salt solution 1 M without previous addition of the acid solution. The sample was sonicated for 1 min and magnetically stirred for 48 h. Afterwards the nanofluid was titrated with NaOH 0.1 M. Finally, the IEC was calculated as follows:

4 $${n}_{{H}_{disp}^{+}}={V}_{NaOH}\cdot {M}_{NaOH}$$

where ${n}_{{H}_{disp}^{+}}\,$are the moles of H^+^ directly displaced from the nanoparticle surface by ion exchanged groups to the aqueous media and *V*_*NaOH*_ and *M*_*NaOH*_ are the volume and concentration of the NaOH used in the titration respectively.

5 $$IEC=\frac{{n}_{{H}_{disp}^{+}}}{{m}_{np}}$$

The experimental error was calculated by means of propagation of uncertainties of the balance, pipettes and burettes used to do the tests.

### Chemical composition and functionalization of nanoparticles

The changes in the chemical composition of the nanoparticles after being isolated from the salt were observed by means of Fourier-transform infrared spectroscopy (FTIR). Tests were performed by a FT/IR-6200 (Jasco) spectrometer with a spectral window of 4000–400 cm^−1^ in transmission mode. A small amount of sample (~1 wt%) was mixed with KBr (IR spectroscopy grade, Scharlab SL), ground down and pressed into pellets of 13 mm of diameter.

### Specific heat capacity

**Figure 10:**
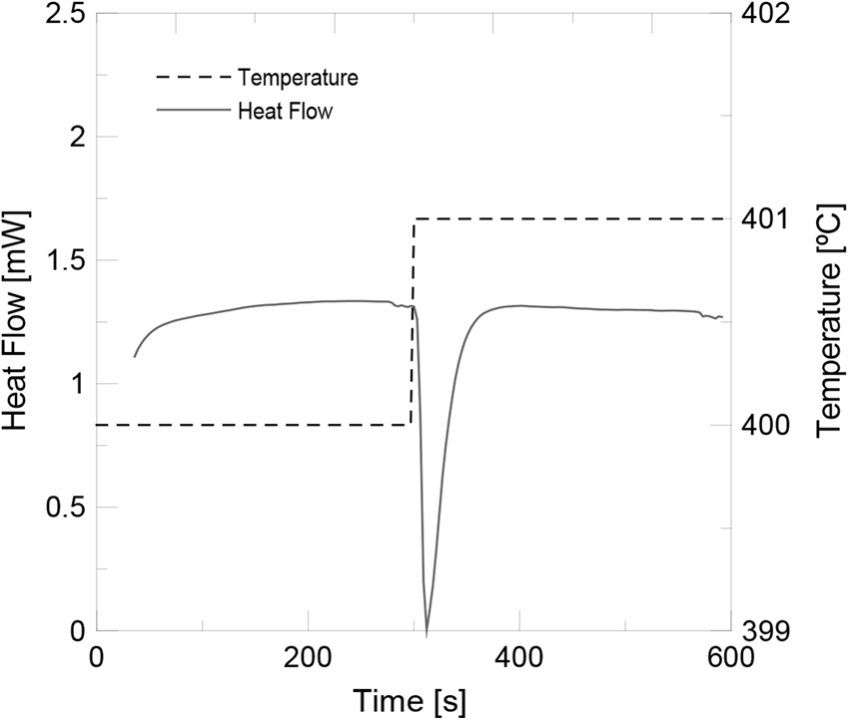
DSC method for specific heat measurement.

The specific heat capacity of salt based nanofluids and isolated functionalized nanoparticles was measured by means of differential scanning calorimetry (DSC) with a Mettler Toledo DSC2 calorimeter. The method used was the areas method that has been checked to provide better results than the dynamic or isostep methods^37^. In this method, a standard sapphire and the sample were submitted to consecutive isothermal segments for 5 min each one with no heating stages amid, with isotherms of 400 °C and 401 °C, respectively. In the 1 °C step, the DSC signal provided a peak (see Fig. 10), whose area was used to calculate the specific heat as follows:

6 $${A}_{S}={c}_{P,S\_exp}\cdot \beta $$

7 $${A}_{m}={c}_{P,m\_exp}\cdot \beta $$

8 $$\alpha =\frac{{c}_{P,S\_real}}{{c}_{P,S\_exp}}=\frac{{c}_{P,S\_real}}{{A}_{S}/\beta }$$

9 $${c}_{P,{m}_{real}}=\alpha \cdot {c}_{P,m\_exp}=\alpha \cdot ({A}_{m}/\beta )$$

where *A*_*S*_ and *A*_*m*_ are the integrated peak area normalized per mass unitfor the sapphire and the sample respectively, ${c}_{P,S\_real}$ is the real value for the specific heat of sapphire found in the literature, ${c}_{P,S\_exp}$ is the experimental specific heat for sapphire, ${c}_{P,m\_real}$ is the real value for the specific heat of the sample, ${c}_{P,m\_exp}$ is the experimental specific heat for the sample, *α* is the correction factor between the experimental and real values and *β* is the temperature step (1 °C).

Standard 40 mL aluminium crucibles were used in this study. To ensure repeatability four samples of 10 mg for nanoparticles and 20 mg for salt based nanofluids were prepared. For each one, two cycles were run in order to obtain a mean value of at least eight different results for the specific heat at 400 °C. The experimental error of the mean value was statistically obtained at a 95% of confidence level, with a mean error of 4.6%. Tests were carried out at a constant 25 mL/min N_2_ flow rate.
